# ORCHIDS: an Observational Randomized Controlled Trial on Childhood Differential Susceptibility

**DOI:** 10.1186/1471-2458-12-917

**Published:** 2012-10-29

**Authors:** Rabia R Chhangur, Joyce Weeland, Geertjan Overbeek, WalterCHJ Matthys, Bram Orobio de Castro

**Affiliations:** 1 Department of Developmental Psychology, Utrecht Centre for Child and Adolescent Studies, Utrecht University, PO Box 80.140, Utrecht, 3508 TC, The Netherlands

**Keywords:** Randomized controlled trial, Externalizing behavior, Child behavior, Parenting, Gene-environment interaction, Differential susceptibility

## Abstract

**Background:**

A central tenet in developmental psychopathology is that childhood rearing experiences have a major impact on children’s development. Recently, candidate genes have been identified that may cause children to be differentially susceptible to these experiences (i.e., susceptibility genes). However, our understanding of the differential impact of parenting is limited at best. Specifically, more experimental research is needed. The ORCHIDS study will investigate gene-(gene-)environment interactions to obtain more insight into a) moderating effects of polymorphisms on the link between parenting and child behavior, and b) behavioral mechanisms that underlie these gene-(gene-)environment interactions in an experimental design.

**Methods/Design:**

The ORCHIDS study is a randomized controlled trial, in which the environment will be manipulated with an intervention (i.e., Incredible Years parent training). In a screening, families with children aged 4–8 who show mild to (sub)clinical behavior problems will be targeted through community records via two Dutch regional healthcare organizations. Assessments in both the intervention and control condition will be conducted at baseline (i.e., pretest), after 6 months (i.e., posttest), and after 10 months (i.e., follow-up).

**Discussion:**

This study protocol describes the design of a randomized controlled trial that investigates gene-(gene-)environment interactions in the development of child behavior. Two hypotheses will be tested. First, we expect that children in the intervention condition who carry one or more susceptibility genes will show significantly lower levels of problem behavior and higher levels of prosocial behavior after their parent(s) received the Incredible Years training, compared to children without these genes, or children in the control group. Second, we expect that children carrying one or more susceptibility genes will show a heightened sensitivity to changes in parenting behaviors, and will manifest higher emotional synchronization in dyadic interchanges with their parents. This may lead to either more prosocial behavior or antisocial behavior depending on their parents’ behavior.

**Trial registration:**

Dutch Trial Register (NTR3594)

## Background

A central tenet in developmental psychopathology is that childhood rearing experiences have a major impact on children’s development across life [1]. At the same time, we know that not all children are equally susceptible to these experiences [2]. Grounded in a diathesis-stress model, there has been growing attention for research on individuals’ genetic susceptibility to parenting. The diathesis-stress model holds that some children, due to a specific vulnerability, are more likely to be negatively affected by environmental risk, such as with parental harshness, than others [3-5].

A typical characteristic of these studies is that they only examined environmental adversity and negative child outcomes. It may therefore be that we, for a long time, only studied so-called *dandelions*; the resilient children that do well even in the face of severe adversity [6]. In doing so, we may have overlooked the *orchids*; children who will suffer severely if ignored or maltreated, but flourish spectacularly when receiving adequate care. This metaphor forms the basis of an intriguing alternative hypothesis, namely the differential susceptibility hypothesis which holds that some children, due to a specific susceptibility factor, are more likely to be affected by environmental factors, *for better and for worse*[7,8].

Preliminary evidence for this differential susceptibility hypothesis has accumulated over the past years. Previous studies demonstrated, for example, that children with the dopamine receptor D4 (DRD4) 7-repeat allele showed significantly more externalizing problem behavior when mothers were insensitive, but also showed less problem behavior when mothers were highly sensitive, compared to those without the DRD4 7-repeat allele [9]. Studies have identified several candidate genes underlying children’s differential susceptibility (e.g., monoamine oxidase A (MAOA) gene; dopamine transporter (DAT1) gene, dopamine receptor D4 (DRD4) gene, dopamine receptor D2 (DRD2) gene, serotonin-transporter (5-HTTLP-R) gene, and the catechol-o-methyltransferase (COMT) gene [10-15].

However, the tenability of the genetic differential susceptibility hypothesis is still unclear, for several reasons. First, most previous studies only measured the presence or absence of environmental adversity and developmental problems, but not environmental enrichment and children’s competence. The absence of parental maltreatment, however, is not the same as parental warmth or sensitivity [16]. Only environmental conditions and outcomes ranging from dysfunction to competence make it possible to avoid ceiling effects in testing differential susceptibility [7,17,18]. Most importantly, most previous studies used correlational designs [19] and therefore alternative explanations for gene-environment (G × E) interactions cannot be ruled out. For example, children with oppositional behavior may be, genetically, more likely to evoke harsh parental discipline and to actively select environments that support their problem behavior.

Trials in which families are randomly distributed across different environmental conditions offer a solution to this problem [20,21], because they permit a manipulation of the environment that is *independent* of children’s genetic makeup and developmental histories. To our knowledge four randomized controlled trials on G × E interactions in children’s social emotional development have been conducted so far [17,22-24].

These pioneering studies delivered important new insights, however their impact suffered from limitations as well. First, although the trials measured environmental enrichment the outcome was usually measured as a decrease in adolescent and child problem behavior, overlooking a possible increase in competent behavior. However, as argued above, to adequately examine differential susceptibility measurements of both environment and child behavior should range from dysfunction to competence. Second, the trials did not examine the possible underlying behavioral mechanism through which a G × E interaction may lead to different behavioral outcomes. It may be that carriers of candidate susceptibility genes show heightened behavioral reactivity. For example, children with low levels of dopaminergic functioning, associated with low reward sensitivity [25], may improve more during and after parent management training than those with high levels of dopaminergic functioning associated with high reward sensitivity, because they can benefit more from the individualization of use of rewards and extensive praising by parents. Likewise, children with decreased serotonergic functioning [25], associated with negative affect/mood, may improve strongly during and after parenting training due to the effect of an increase in positive parental emotions on this affect/mood. A highly reactive child will likely show an intense, mirroring emotional response to both negative and positive discipline [27,28], which, in turn may lead to emotional synchronization in parent–child interactions. This congruency in affect may then lead to the development of either problem or prosocial behavior, depending on either positive or negative interactions with parents. Therefore, research should also investigate genetic expression “outside the skin”: the mechanisms through which genetic variation moderates the impact of environmental influences on individuals’ development. A randomized controlled trial can test hypotheses about underlying behavioral processes by examining whether certain mechanisms change in the experimental condition, mediating the intervention effect [29].

### Aim and hypotheses

The ORCHIDS study is a genetically informed randomized controlled trial to examine possible G × E and G × G × E (i.e., polygenetic) interactions in the development of child behavior. The study examines parenting in its full scope, from both harsh and inconsistent to positive, sensitive, and appropriate parenting behavior as well as from children’s problem behavior and difficulties to their skills, competencies, and strengths. The primary aim is to investigate whether enrichment of the environment, based on the Incredible Years (IY) parent training, has more effect on a genetically susceptible subgroup of children, and to investigate why this may be the case. We expect that the parent training will bring about an environmental enrichment, leading to behavior changes in the participating parents. Two hypotheses will be tested. First, we expect differential susceptibility, which means that children in the intervention condition who carry one or more susceptibility genes (i.e., carrying a MAOA low activity (short) allele; DAT 10-repeat allele; DRD4 7-repeat allele; DRD2 A1 allele; 5-HTTLPR short allele; and or a COMT val allele) will show a significantly higher decrease of problem behavior and increase of prosocial behavior after their parent(s) received the parent training, compared to children without such susceptibility genes and children in the control group. In the control group, we expect this same genetic subgroup to show most behavior problems and least prosocial behavior. Second, we expect that emotional synchronization in parent–child interactions will mediate the intervention effect. Specifically, we expect that children who carry one or more susceptibility genes show a higher synchronization to their parents’ affect than children without these susceptibility genes. Therefore, we expect these children to benefit most from the increase in parental positive affect and sensitivity induced by the Incredible Years intervention.

## Methods/Design

### Design

The ORCHIDS study is a randomized controlled trial with an intervention (i.e., the Incredible Years parent training) and a control condition that tests gene-based differential susceptibility to changes in parenting. Participants will be 480 families, with children aged 4–8 who show mild to (sub)clinical externalizing behavior problems. Of those families, 160 will be randomly assigned to the intervention condition and 320 families to the control condition. After enrollment in the trial and randomization, the baseline assessment (pretest) will be carried out. The Incredible Years (IY) program will be implemented after these baseline assessments. Participants in the control condition will receive no intervention, but are allowed –and, in case needed, are assisted– to seek mental health care and parenting support through regular services. Posttest and follow-up assessments will be conducted after 6 months and after 10 months, respectively. Approval for data collection was obtained from the central committee on research involving human subjects in The Netherlands (METC UMC Utrecht, protocol number 11-320/K).

### Recruitment

**Figure 1:**
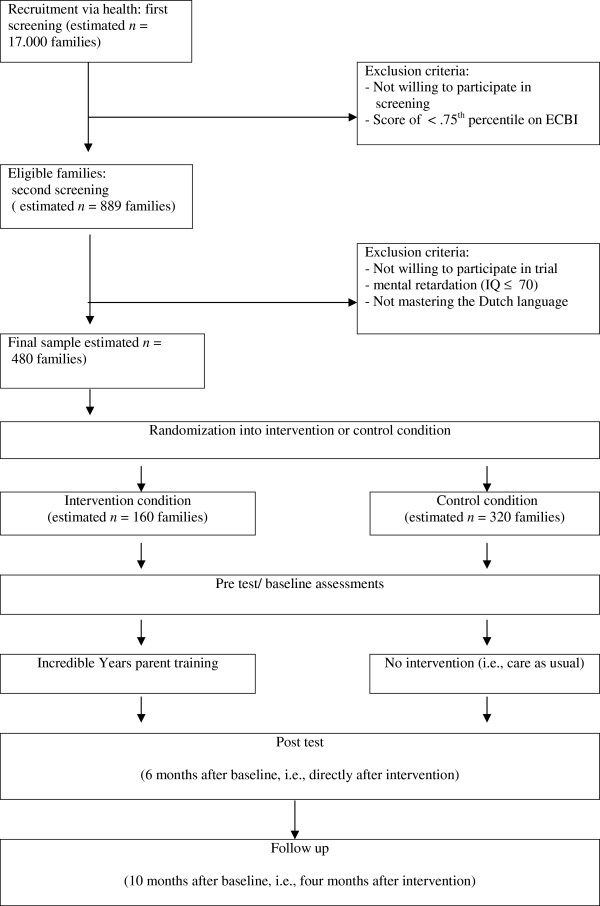
Study design (CONSORT schedule).

In a first screening (see Figure 1), roughly 17.000 families will be targeted through community records via two Dutch regional health care organizations (estimated response rate is 52%, see [30]). All families will receive a personalized information letter, including the Eyberg Child Behavior Inventory (i.e., ECBI) to screen for children’s problem behavior [30]. The criterion for inclusion will be a score at or above the 75^th^ percentile. This cut-off is chosen so that at-risk families will be selected, without excluding children and parents with subclinical or even normal-range functioning. Based on a conservative estimation, 889 families (10%) are expected to be eligible for inclusion. Additionally, a second screening will take place to check for exclusion criteria: mental retardation of the parent and/or child (IQ ≤ 70) and not mastering the Dutch language. Based on estimates from similar procedures followed in previous research [31], 480 eligible families are expected to eventually participate in the ORCHIDS study. These families will receive a second invitation letter and will be contacted for trial participation.

### Randomization

Participants will be informed of the design of the study and will give consent prior to randomization. Randomization will occur through random selection of a participant number that is linked to either the intervention or control condition.

### Sample size calculation

Power analyses are essential to maximize chances to find significant G × E interactions [32]. For this calculation, we used a Fixed Effects ANOVA Power Analysis in PASS11 [33]. Based on a meta-analysis that demonstrated a small effect size (*d)* of .20 of the IY intervention [34], and assuming a small G x E interaction effect and no main effects of genes [3,35,36], a sample size of *N* = 480 families will be required for investigating our hypotheses in a two-sided test at α = .05 and power (1-β) = .80.

### Intervention

The IY training is aimed at improving parenting skills in order to reduce child behavioral problems, such as aggressive behavior, and enhance competent behavior. The IY training includes 14 to 16 weekly two hour sessions. During these sessions parents watch video-vignettes, discuss parenting with each other and practice new techniques in role-plays. Each group will consist of approximately 10 to 12 parents. IY parent training is different from most other parent (management) training programs, in that trainers use a collaborative leading style: They do not instruct, but are part of the group and lead discussions [3,37,38]. Many previous randomized trials have shown the program to be effective [39], also in The Netherlands [40]. Hence, IY parent training is an evidence-based parent training.

### Data collection

An overview of all measurement occasions is given in Table 1. Both the recruitment, as well as the waves of data collection will be conducted in two separate cohorts in 2012/2013 and 2013/2014. The recruitment (i.e., screening) will take place in September-October 2012 and 2013. The pretests will take place in November-January 2012 and 2013. Families will be asked to fill in questionnaires in the presence of a researcher or research assistant during home visits. Furthermore, parent–child interactions will be videotaped during structured play situations. This procedure will be repeated twice after the pretest, namely at posttest and follow-up. Saliva samples for genotyping will be collected once during pretest.

**Table 1:** Overview of measurements

**Measurement**	**Screening**	**Pre test**	**Post test (6 months after baseline)**	**Follow up (10 months after baseline)**
Demographic characteristics		*		
Child behavior:				
External behavior (ECBI)	*	*	*	*
Prosocial behavior (MESSY)		*	*	*
Temperament: (CBQ)		*	*	*
Strengths and Difficulties (SDQ)		*	*	*
Genotyping *(DRD4, DRD2, DAT1, COMT, 5-HTTLPR, MAOA)*		*		
Parenting behavior:				
PPI		*	*	*
Parent-child interaction:				
DPICS-R		*	*	*

*Note*. *ECBI* Eyberg Child Behavior Inventory, *MESSY* The Matson Evaluation of Social Skills with Youngsters, *CBQ* Children’s Behavior Questionnaire, *SDQ* Strengths and Difficulties Questionnaire, *DRD4* dopamine receptor D4 gene, *DRD2* dopamine receptor D2, *DAT1* dopamine transporter gene, *COMT* catechol-o-methyltransferase gene = *5-HTTLPR* = serotonin-transporter gene, *MAOA* monoamine oxidase A gene, *PPI* Parenting Practices Interview, *DPICS-R* Dyadic Parent-Child Interactive Coding System-Revised.

### Outcomes

Primary outcomes are the possible moderating effects of child genotype on the IY intervention effect (i.e., on the decrease in externalizing problems behavior and/or increase in prosocial behavior of the child). The intervention effect will be assessed with the ECBI, the Matson Evaluation of Social Skills with Youngsters (MESSY [41]), and the Dyadic Parent–child Interaction Coding System-Revised (DPICS-R [42,43]), see measures in Table 1. Secondary outcomes will be the observed (changes in) emotional synchronization in parent–child interactions as possible underlying behavioral mechanism to the G × (G) × E interactions.

### Analyses

First, using independent *t*-tests, we will examine whether randomization was successful, comparing baseline levels of externalizing and prosocial behavior across the intervention and control condition. Possible significant differences at baseline will be used as covariates in analyses [44]. Second, as the longitudinal data of individuals will be nested in families, multilevel latent growth curve analyses in Mplus [45] will be performed. In this analysis, we will test the interaction between the variables ‘group’ (i.e., intervention vs. control) and ‘genotype’ (i.e., susceptible genotype vs. non-susceptible genotype) to examine possible G × E interactions. In addition, we will take into account families’ ethnic background (i.e., Caucasian vs. non-Caucasian) and additional parental support or (mental) health care families received during the study (i.e., additional self sought care vs. no additional self sought care). Ethnic background and additional care will both be used as covariates in the analyses.

## Discussion

The ORCHIDS study described in this protocol is a genetically informed randomized controlled trial targeting families with children aged 4–8 who show mild to (sub)clinical behavior problems. The primary aim of ORCHIDS is to assess possible G × (G×)E interactions in the development of child behavior in its full scope – that is, from children’s problem behavior to their competencies. Our large scale randomized controlled trial is one of the first experimental studies of G × E interactions in social development. Experimental manipulation of the environment is crucial in understanding G × E interactions, because it is the only way to prevent confounding gene-environment covariation.

Additional to a single gene approach, we will investigate possible cumulative effects of multiple candidate genes (i.e., G × G × E or polygenetic interactions [46]). Also, we will make a first attempt to obtain more insight into the behavioral mechanism underlying these G × E interactions by examining (changes in) emotional synchronization in observed parent–child interactions.

Better insight into individual differences in, for example, reactivity to positive parenting behavior like praise, associated with dopaminergic and serotonergic functioning, may help improve the tailoring of behavioral parent training. This seems necessary as the mean effect size of these interventions is modest (Cohen’s *d* = 0.47 [47]). Individualization of use of rewards and praise may help increase the efficiency of these parenting skills. For example, if the emotional significance of the positive message of praise is less well processed, associated with altered dopaminergic functioning, both verbal and nonverbal enthusiasm may be particularly relevant for this specific subgroup of children [26]. Thus, instead of delivering interventions in a standardized way, parenting programs may benefit from an individualized approach based on insights from results of studies like the present one.

Despite the strengths and innovative aspects of ORCHIDS, there are some issues that our study is unable to take into account. Differential susceptibility to parenting may also be caused by environmental influences that alter the effects of genes (i.e., epigenetics), rather than by specific DNA sequences or a certain number of repeats alone [48]. Human development is an active process powered by a continuous interaction between the genome and the environment [49]. DNA methylation (i.e., the biochemical process that involves the addition of a methyl group onto cytosine in the DNA, regulating the operation of the human genome), for example, has been shown to mediate the relation between genotype and developmental outcomes [50,51].

Once differential susceptibility to the environmental manipulations has been demonstrated, a next step will be to further investigate the behavioral as well as neurobiological underlying mechanisms of genetic differences in sensitivity to change. Interpreting the intervention effect in this study will be like looking at an “*omnibus* effect” that covers a variety of possible environmental effects or change mechanisms; the environmental change induced by the intervention consists of changes in many different parent behaviors and child responses. Which of these changes are driving the omnibus effect cannot be elucidated in a RCT. In order to create a more complete picture of gene-environment interplay, multiple genetically informed experimental designs should be used additionally to large scale RCT’s such as microtrials: small-scale, randomized experiments using a brief and focused environmental manipulation, designed to suppress specific risk mechanisms or enhance specific protective mechanisms but not to bring about full treatment or prevention effects in outcome [52].

## Conclusion

The ORCHIDS study will investigate possible G × (G×)E interactions in the development of both positive and negative child behavior by assessing whether an experimental manipulation of the environment with the Incredible Years intervention is more effective for a particular genetic subgroup of children than for others. With this study we will contribute to a further understanding of moderating effects of specific alleles (i.e., polymorphisms) on the malleability of child behavior, and the behavioral mechanisms that may underlie gene-environment interactions. By doing so we gain more insight into what works for whom and how it works when it comes to interventions targeting child problem behavior.

### Trial status

The trial is ongoing, still recruiting participants.

## Abbreviations

5-HTTLPR: Serotonin-transporter gene; DAT1: Dopamine transporter gene; DPICS-R: Dyadic Parent–child Interactive Coding System-Revised; DRD2: Dopamine receptor D2; DRD4: Dopamine receptor D4 gene; COMT: Catechol-o-methyltransferase gene; ECBI: Eyberg Child Behavior Inventory; G × E: Gene by environment interaction; G × G × E: Gene by gene by environment interaction; IY: Incredible Years; MAOA: Monoamine oxidase A gene; MESSY: The Matson Evaluation of Social Skills with Youngsters; RCT: Randomized Controlled Trial.

## Competing interests

The authors declare that they have no competing interests.

## Authors' contributions

GO designed this study and reviewed and revised the manuscript. RC and JW reviewed the design and drafted the manuscript. WM and BOC reviewed and revised the manuscript. All authors have read and approved the final manuscript to be published.

## Pre-publication history

The pre-publication history for this paper can be accessed here:

http://www.biomedcentral.com/1471-2458/12/917/prepub
